# Small philanthropy and big science: the RETROVIROLOGY prize and Stephen P. Goff

**DOI:** 10.1186/1742-4690-2-43

**Published:** 2005-07-06

**Authors:** Kuan-Teh Jeang

**Affiliations:** 1 The National Institutes of Health, Bethesda, MD, USA

## Abstract

Stephen P. Goff wins the 2005 RETROVIROLOGY prize.

Earlier this year, I announced that *Retrovirology *would inaugurate an annual prize to recognize the achievements of a deserving mid-career retrovirologist [1]. There are two reasons why we feel such an award is timely. First, retrovirology is an increasingly robust and important field of science. It seems wanting that no award exists to recognize exclusively excellence in basic retrovirus research. Second, although there are many scientific prizes and awards, very few are targeted only to mid-career scientists. In principle, the awarding of a prize carries two worthy aims. Prizes reward individuals for past achievements, and prizes also bring visibility and encourage future advances in particular research problems/fields. For the latter goal, choosing an outstanding mid-career individual who is on the peak of his/her productivity curve and who is poised for many years of "best" work ahead is more than appropriate. Hence, while the roster of winners of many awards frequently reads the same as other awards, the RETROVIROLOGY prize intends to highlight fresh scientists for their ongoing and accelerating research achievements.

A few wealthy international philanthropies frequently and deservedly capture the limelight with their research largess. On the other hand, every department Chairman and Dean of schools recognize the unsung importance of the many small local donors who fund the student scholarships, the travel stipends, and the stop-gap institutional research grants that keep the daily lifeblood of science flowing. The inaugural RETROVIROLOGY prize is supported through a donation from the Ming K. Jeang Foundation, an educational foundation based in Houston, Texas, which has provided for scholarships at Houston schools, at the University of Arizona, and at the Johns Hopkins University School of Medicine. Our inaugural award is named the M. Jeang RETROVIROLOGY prize.

For 2005, the Editors of *Retrovirology *selected Stephen P. Goff as the first recipient of the RETROVIROLOGY prize. Stephen Goff is the Higgins Professor of Biochemistry and Molecular Biophysics at the College of Physicians and Surgeons, Columbia University, USA. He was one of the first investigators to clone a functional copy of a retroviral genome, and to use recombinant DNA methods to study viral replication. Over the last two decades Dr. Goff has developed and exploited the Moloney murine leukemia virus as a genetic system. One of his most important results was the definition of the functional domains of the viral *pol *gene, and, especially, the seminal discovery of a viral function, now termed the integrase function, required for the integration of viral DNA into the host genome. Dr. Goff was also the first to develop plasmids that express enzymatically active reverse transcriptase (RT) of both mouse and human viruses. Fine-structure mutational analysis proved that reverse transcriptase contains separate, and separable, DNA polymerase and RNAse H domains.

Dr. Goff has used the yeast two-hybrid system to detect and characterize protein-protein interactions between viral proteins, and also between viral and host gene products. He discovered that cyclophilin A, a host prolyl isomerase, binds specifically to the HIV-1 Gag, and regulates both infectivity and escape from host restrictions. Dr. Goff has additionally pioneered the use of somatic cell genetics to identify host genes that affect retrovirus replication. He has identified a novel zinc-finger antiviral protein (ZAP) which directly binds specific sequences in viral RNAs in the cytoplasm and targets these RNAs for degradation.

Goff has also been active in the study of retroviral oncogenes, notably the v-*abl *gene carried by the Abelson murine leukemia virus. Goff was the first to prepare molecular clones of the viral genome and the cellular protooncogene, and to map the structure and chromosomal location of c-*abl*. The Goff laboratory has identified several novel Abl-binding proteins that regulate Abl signalling. He has shown that one such bridging molecule, PST-PIP1, directs phosphatases to act on Abl and thereby negatively regulate its tyrosine kinase activity.

In choosing the prize winner, the Editors considered more than just scientific excellence, and sought to identify the rare individual who is both an outstanding researcher and a selfless mentor. In the big picture of science, the successes of a scientist's students and students-of-students in generations hence likely hold much larger impact than even the most stellar singular career. Fittingly, at mid-career, Stephen Goff has already trained several highly successful scientists.

To understand Steve better as a scientist and a teacher, I had an opportunity in a question-and-answer session to solicit his views on several issues of interest. His answers to my questions are presented below.

KTJ: Did you always want to be a scientist or was there something else that you wanted to do when you were young?

SPG: I knew I wanted to do science very early in life, beginning with grade school. My father was a contractor, carpenter, boat-builder, mechanic, and clock repairman, and had a fabulous workshop. I grew up taking machinery apart and putting it back together and have always enjoyed watching complicated machines at work. I was particularly fascinated by chemistry in high school and with the idea that molecules were just very small machines. My older brother Chris went into molecular biology before me, and it was completely natural to me to follow in his footsteps. I'm still fascinated with these molecular machines that support our lives.

KTJ: What attracted you to go into retrovirus research?

SPG: I was first attracted to work on the small DNA viruses at Stanford as a graduate student in Paul Berg's lab because of the amazing techniques to manipulate DNA that were just being developed – SV40 provided the first chance to isolate and manipulate the complete genome of a simple organism (even before recombinant DNA). I liked the notion that one could write or modify a genetic program, much as one could write a computer program, and watch the results in action. As I looked around for possible subjects of study for my postdoc, I was very attracted by the RNA tumor viruses and their complex life cycle, at that time only known in outline, and their potential for gene therapy. A meeting with David Baltimore in 1977 convinced me that his lab would be an amazing place to apply recombinant DNA methods to the study of these viruses, and this cemented my choice. I've been working on these viruses ever since.

KTJ: Who had the most influence on your career and how?

SPG: Many people have shaped my thinking and the direction of my research. Paul Berg taught me how to design experiments, how to think carefully and fully about data, and how to present results clearly in writing and at meetings. David Baltimore taught me how to be incisive and efficient at work, and to focus on the most important issues – and to be bold. Many people in the field have been and continue to serve as role models: Howard Temin, Frank Lilly, Harold Varmus, and John Coffin have been particularly important.

KTJ: What do you see as some of the important questions in retrovirology in the near and long term future?

SPG: I think one obvious growth area is the interaction of viruses with the host. New tools – mainly genetic ones – have made it possible to identify and characterize the interactions of viral proteins with specific cellular machinery, and to learn how the viruses exploit that machinery for intracellular movement, macromolecular synthesis, and assembly. Another related area is the innate or intrinsic immunity to retrovirus infection that is just now being uncovered. I think one of the original justifications for the study of viruses – their ability to spotlight exciting or critical aspects of cell biology – is now being powerfully validated.

KTJ: What do you consider as your most important scientific contribution, and what is currently the most exciting research in your laboratory?

SPG: Broadly, I would suppose that my major contribution has been the development of the murine leukemia virus as a genetic system. Within that area, I think the characterizations of viral mutants defective in the protease, the integrase, and p12 gag are likely the most important results, both for their implications for basic virology and for the applied aspects of antiviral drug development. I am also proud of cloning the v-abl oncogene and c-abl protooncogene, which has ultimately led to the antitumor drug Gleevec, and of helping Liz Robertson in the development of mouse knock-out technology used in the isolation of abl KO mice.

KTJ: Young scientists who are yet established can sometimes feel that their manuscripts and/or their grants have been unfairly reviewed. What is your advice to them?

SPG: Many of us, young and old, often feel that our work has been unfairly reviewed and deserves reconsideration. It is important to develop a thick skin and keep trying – both in terms of resubmissions and in submitting to alternative journals. I encourage budding authors to rebut negative reviews as directly as possible and to request that the editors stand in judgement of the issues. When those avenues fail for a given journal, I would encourage young scientists to move on to the next logical journal, and given the expanding number of new journals there is little chance that an important result will go unpublished through prejudice or bias. I have great faith in the peer review process. In terms of grant reviews, competition is now at an almost unprecedented high. For new investigators working to establish their laboratories, this is a very difficult time to find funding, but here too persistence is key. Don't be discouraged and keep applying – quality will be rewarded.

KTJ: There are great scientists who do great science, and then there are great scientists who do great science and train great scientists. What are your secrets to training great scientists?

SPG: I hope that I will be remembered as someone who trained a large number of active and productive scientists – at last count I have graduated 25 students and trained about the same number of fellows. I try to give my students considerable freedom to explore new avenues, to fail and succeed, and so to learn by experience what is worthwhile and what is too risky. I try to encourage optimism, self-motivation, and give some sense of the excitement we all have in what we do.

KTJ: If one remembers you by the scientists that you have trained, who would you wanted to be remembered by and why?

SPG: I suppose one is most attached to the earliest people in the lab, those who helped establish us as an ongoing concern. In my case, the list would have to include such early students as John Colicelli, Naoko Tanese, and Pam Schwartzberg, and my first postdoc Monica Roth, all of whom are running active labs today. All are superb scientists and I consider myself blessed to have had them working with me – I would be proud to be remembered as having helped train them. But any such list is wildly incomplete, and I feel the same way about everyone who has passed through my laboratory. All are like family.

KTJ: How do you see your role with your former trainees after they have left your lab?

SPG: I feel it is critically important to set my outgoing people on a productive course, with at least one project for the fellows immediately ready to go – with the hope of quick and easy experiments. The pressure for rapid publications from a new laboratory is so intense today that successful trainees need to hit the ground running and do meaningful experiments from their first day in their new home. We have established a pattern where outgoing fellows are given the opportunity to exploit a new gene that they have identified in my lab and can work to develop an understanding of its mode of action, its partners, and its significance in the life cycle. I am always thrilled to read new papers from my former trainees as they expand the boundaries of virology.

KTJ: Someone told me once that every 10 years he would like to reinvent himself. What do you see yourself doing 10 years from now that might be different from what you are doing today? When do you think you would stop doing research and how will you know when the time is right?

SPG: I would be perfectly happy to be doing science ten years from now in much the same way I am now – realizing that the actual science we do is changing every day. The great thing about our field is how rapidly technology opens new doors and expands our capabilities, and I like to be early in applying new methods. I think I will stop running a laboratory when I no longer feel able to contribute to technology development, to effectively apply new methods to problems, and to be nimble. My students will probably let me know.

KTJ: In my office there is a quotation by Edmond Burke 'All that is necessary for the triumph of evil is that good men do and say nothing'. If there is one thing that you think needs to be said or done in science (or in society at large), what would that be?

SPG: A major problem for scientists is that the power and contributions of science to our society are grossly underappreciated by the nonscientific public at large. The gap between scientists and nonscientists is growing wider and is undercutting support for our work. We must fight this trend and point out to Congress all the benefits of their support. We as scientists must try to communicate the value and excitement of our work to our nonscientific friends.

KTJ: Would you want your children to become basic research scientists? Why or why not?

SPG: I think this is a wonderful time to enter the field of basic research, with the most amazing opportunities for new findings just coming available – even if funding in the next few years looks to be tight. But being a successful basic scientist requires a love of experimentation and an innate drive to learn about the way things work, and it is a demanding life. Neither of my two children is inclined toward basic science, and without that natural bent I would never push them into my field. I want them to be successful and happy in whatever way they choose to contribute to society.

KTJ: With the understanding that it will be a long time in the future, what would you like your headstone to read?

SPG: Maybe "He made it work;" or "He fixed it;" or"He liked solving puzzles."

**Figure 1:**
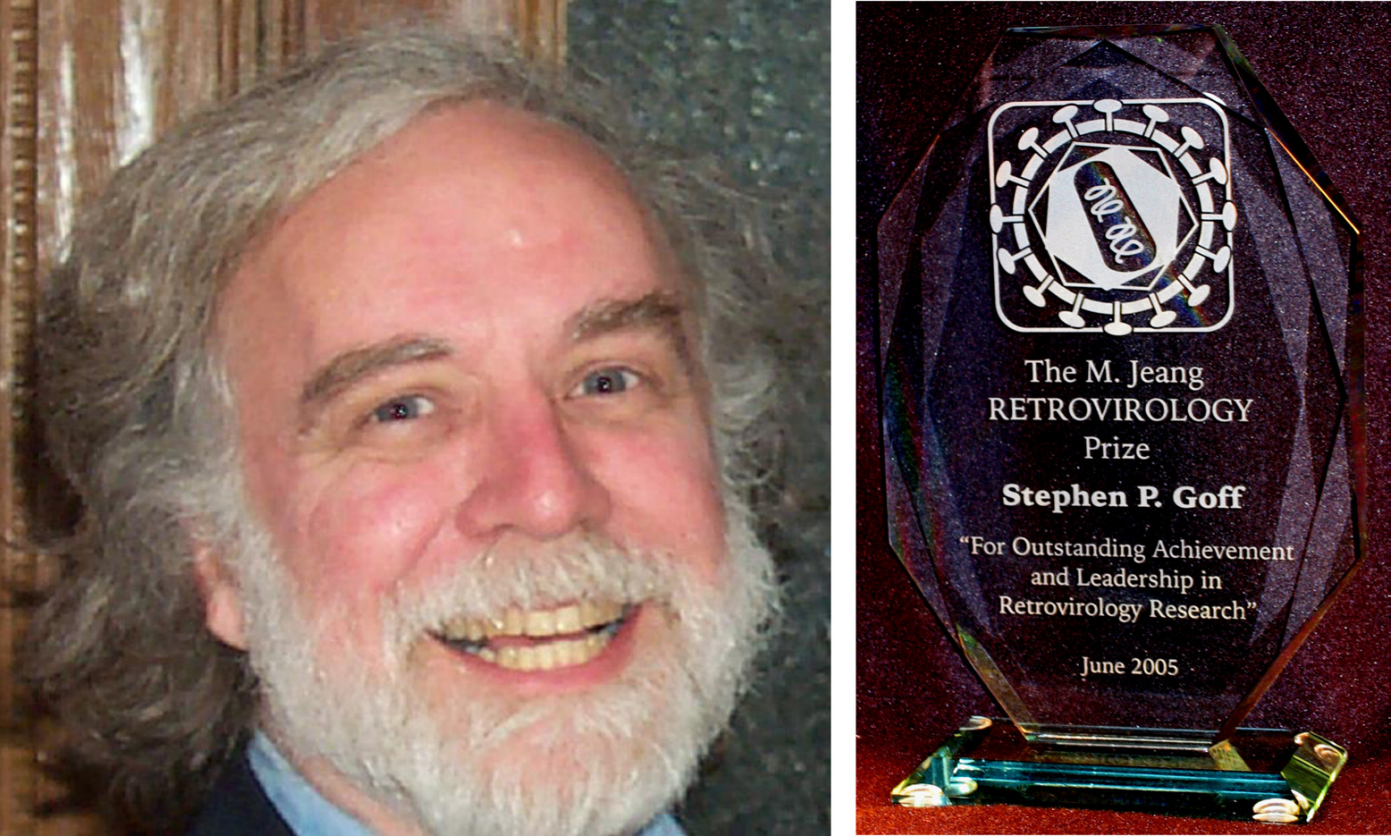
Left, photograph of Stephen P. Goff; right, an engraved crystal trophy and a cash honorarium were presented to Dr. Goff in honor of his selection as the first RETROVIROLOGY prize recipient.
